# Clinical value of computed tomography in suspected prosthetic valve dysfunction

**DOI:** 10.1007/s12471-026-02052-8

**Published:** 2026-07-02

**Authors:** Marco Voortman, Clemens von Birgelen, Daan Ties, Tomasz Plonek, Alexander R. van Rosendael, Carine J. M. Doggen, Lodewijk J. Wagenaar

**Affiliations:** 1 Department of Cardiology, Thoraxcentrum Twente, Medisch Spectrum Twente, Koningsplein 1, 7512 KZ Enschede, The Netherlands; 2 Department of Health Technology and Services Research, Faculty of Behavioral Management and Social Sciences, Technical Medical Center, University of Twente, Enschede, The Netherlands; 3 Department of Radiology, Medisch Spectrum Twente, Enschede, The Netherlands; 4 Department of Thoracic Surgery, Thoraxcentrum Twente, Medisch Spectrum Twente, Enschede, The Netherlands

**Keywords:** Computed tomography, Prosthetic heart valve, Structural valve degeneration

## Abstract

**Background:**

Prosthetic valve dysfunction (PVD) is potentially life-threatening, yet often challenging to diagnose. We aimed to determine the negative and positive predictive value (NPV, PPV) of cardiac computed tomography (CT) in clinically suspected PVD.

**Methods:**

Consecutive patients with prosthetic heart valves (PHVs), who underwent clinically indicated CT for suspected PVD between January 2017 and June 2024, were retrospectively assessed. CT scans with adequate image quality were evaluated and classified (abnormal: periprosthetic masses; structural valve deterioration (SVD); restricted leaflet motion). Valve-related adverse events and follow-up imaging were assessed to evaluate the diagnostic performance.

**Results:**

Of all 64 patients (58.6 ± 16.9 years, 42.2% female), 68.8% had mechanical, 18.8% bioprosthetic, and 12.5% transcatheter PHV. CT findings were *normal* in 34 (53.1%) patients, with an NPV of 94% for ruling out device-related adverse events within a follow-up of 1 year. Among 30 (46.9%) patients with *abnormal *CT findings, a periprosthetic mass was identified in 21 (70%), including pannus (*n* = 10), thrombus (*n* = 6), vegetations (*n* = 3). SVD was observed in 2 patients (6.7%). Among 23 patients with mechanical PHVs, 18 (78.3%) exhibited restricted leaflet motion. Surgical interventions confirmed CT abnormalities in 7/10 patients, yielding a 70% PPV.

**Conclusion:**

This study is the first to demonstrate the high negative predictive value of CT for ruling out significant PVD-related complications, reinforcing its role as a reliable modality for ruling out major adverse events. While the PPV for identifying underlying PVD pathologies was moderate, CT effectively identified structural abnormalities that required intervention, supporting its clinical usefulness for comprehensive assessment of suspected PVD.

**Supplementary Information:**

The online version of this article (10.1007/s12471-026-02052-8) contains supplementary material, which is available to authorized users.

## What’s new?

- This is the first study to show that in patients with clinically suspected prosthetic valve dysfunction, a normal cardiac CT was associated with the absence of adverse events within a 1 year of follow-up, with a negative predictive value of 94%.
- In a cohort largely consisting of mechanical prosthetic valves, CT accurately identified the underlying pathology, with a positive predictive value of 70% confirmed intraoperatively.

## Introduction

Prosthetic valve dysfunction (PVD) is a rare but potentially life-threatening complication after heart valve replacement with a prosthetic heart valve (PHV) [1]. It is associated with significant morbidity and mortality if undiagnosed and left untreated [2, 3]. Detection remains challenging, as patients often present with non-specific symptoms such as dyspnea, fever, or cerebrovascular events and conventional diagnostic modalities may be inconclusive, which impedes clinical decision-making [4].

Transthoracic echocardiography (TTE) is the first-line imaging modality for suspected PVD [5, 6]. Although it can detect an increased transvalvular gradient and an abnormal flow pattern, it may not provide definitive insight into the underlying etiology [7]. Transesophageal echocardiography and fluoroscopy are also commonly used; yet, transesophageal echocardiography is also limited by acoustic shadowing, and fluoroscopy only provides information on leaflet opening angles without revealing the underlying cause [8, 9].

Cardiac computed tomography (CT) has emerged as a complementary imaging tool, enabling assessment of leaflet motion and offering high-resolution anatomic details to identify the underlying etiology of PVD [10, 11]. In fact, CT may provide valuable insight into the mechanism of obstruction, such as pannus, thrombus, vegetation, or structural valve deterioration, thereby facilitating risk stratification and clinical decision-making [7, 11, 12]. While there is consensus about the growing utility of CT in identifying the causes of PVD, its role in definitively excluding major PHV pathology remains unclear.

This study aims to address this gap by evaluating the diagnostic strength of CT in ruling out significant PVD. We assess whether patients with suspected PVD but *no* abnormalities on CT are at low risk for developing severe valve pathologies or PHV-related adverse clinical events. In addition, in patients with CT-positive findings, we assess whether the presumed etiology was confirmed by intraoperative findings.

## Methods

### Study design and patient population

This retrospective cohort study included consecutive adult patients (≥ 18 years) with suspected PVD who underwent CT from January 2017 to June 2024. Suspicion of PVD was based on clinical symptoms (e.g., unexplained dyspnea, fever, cerebrovascular events) or newly elevated transprosthetic gradients during follow-up. Patients were excluded if CT image quality was insufficient or no clinical follow-up was available. Demographic and clinical data were retrieved from electronic medical records. The study was approved by the Institutional Review Board and informed consent was waived due to the retrospective design.

### Echocardiographic imaging

TTE was performed using Philips E33 ultrasound systems. Standardized assessment included transvalvular gradients, effective orifice area, velocity-time integrals, leaflet motion, and presence of regurgitation or masses. For aortic PHVs, a mean transvalvular gradient < 20 mm Hg was considered normal, 20–34 mm Hg suggested possible dysfunction, and ≥ 35 mm Hg indicated a significant stenosis [1]. For mitral PHVs, a mean gradient ≤ 5 mm Hg was considered normal, 6–10 mm Hg suggested possible dysfunction, and > 10 mm Hg indicated significant stenosis [1].

### CT imaging protocol

Cardiac CT was performed using a dual-source CT scanner (SOMATOM Definition Flash, Siemens Healthcare, Forchheim, Germany). CT acquisition was performed at the discretion of the clinical team using a prospective sequential electrocardiogram-gated protocol in three axial stacks, positioning the valve centrally in the middle stack.

Contrast-enhanced acquisition was performed using a dual-phase injection protocol (70 mL bolus contrast followed by 30 mL mixed bolus) with bolus tracking in the descending aorta. Scans were acquired at 120 kVp with dose modulation (CARE Dose4D) and collimation of 128 × 0.6 mm. Reconstructions included: (I) best diastole (32% R‑R); (II) end-diastole (100% R‑R); and (III) dynamic cine (20% to 90% of the R‑R interval in 5% increments). A non-contrast scan was routinely acquired to assess for calcifications.

### Image analysis

Post-processing was performed using the Philips IntelliSpace Portal and images were assessed for leaflet motion and structural abnormalities.

In mechanical PHVs, restricted motion was defined as impaired leaflet movement or a deviation of > 4° from the manufacturer’s reference value [3, 10, 13]. Structural abnormalities included pannus, thrombus, and vegetations, which were differentiated based on anatomical location and morphology, with Hounsfield unit measurements used as supportive information (Fig. S1; [14, 15]). In bioprosthetic PHVs, structural valve deterioration (SVD) was defined as leaflet thickening, leaflet fibrosis, or leaflet injury, with or without calcification. To account for both fibrotic and calcific forms of degeneration, SVD was defined either by marked leaflet thickening or by severe leaflet calcification with an Agatston score ≥ 2,000 AU in men and ≥ 1,200 AU in women [6, 23, 24].

A positive CT finding was defined as the presence of masses, SVD, or restricted leaflet motion. Negative CT findings indicated absence of these abnormalities, with normal leaflet motion.

### Outcome measures

The primary endpoint was a composite of device-related adverse events within 1 year follow-up in patients with negative CT findings, including device malfunction; thromboembolism; endocarditis; valve thrombosis; valve-related mortality; reoperation; or progression of structural valve deterioration (evidenced by a significant increase in transvalvular gradient or reduction in valve area during follow-up).

The negative predictive value (NPV) was calculated based on the event-free survival in CT negative patients. The positive predictive value (PPV) was assessed in patients with positive CT findings, with intraoperative confirmation of structural abnormalities being the reference standard.

### Statistical analysis

Continuous variables were reported as mean ± standard deviation and categorical variables as frequencies and percentages. Differences in echocardiographic findings between CT-positive and CT-negative groups were analyzed using the Monte Carlo exact chi-square test due to small cell counts. A *p*-value < 0.05 was considered statistically significant. Analyses were performed using SPSS version 24.

## Results

**Fig. 1:**
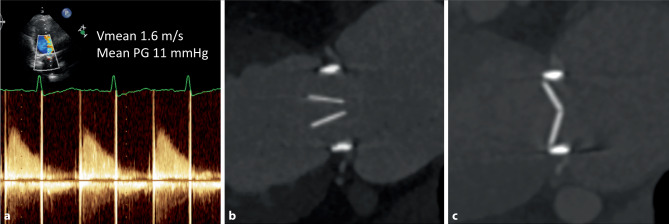
Normal leaflet motion on CT in a patient with elevated Doppler gradient across a mechanical mitral valve prosthesis. Images of a patient with a mechanical PHV (Carbomedics, size 25 mm) who presented with progressive exertional dyspnoea. TTE demonstrated an elevated mean transmitral gradient (11 mm Hg; Panel **a**), whereas transesophageal echocardiography and angiography were unremarkable. Cardiac CT showed normal disc motion without evidence of pannus or thrombus (Panels **b–c**). Dyspnea was attributed to COPD (GOLD II), and conservative management was initiated. At four-year follow-up, the echocardiographic gradient had normalised (3.3 mm Hg) with preserved biventricular function and a stable invasively measured transmitral gradient of 5 mm Hg

**Fig. 2:**
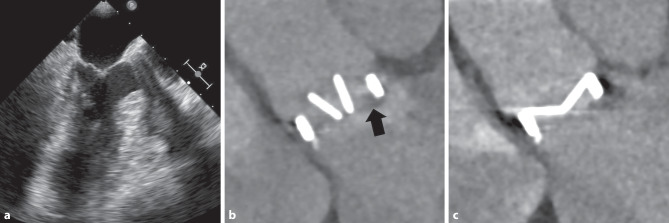
Thrombotic obstruction of a mechanical aortic valve diagnosed by CT. Images in a patient with a mechanical aortic valve (Carbomedics, size 25 mm), who presented with recurrent cortical strokes during episodes of subtherapeutic INR, caused by thromboembolic cerebrovascular occlusion. Transthoracic and transesophageal (panel **a**) echocardiography were inconclusive due to acoustic shadowing from the mechanical PHV, precluding definitive assessment of disc motion. Cardiac CT suggested prosthetic valve thrombosis, showing asymmetric disc opening (67.3 and 79.1°; panel **b**) that indicated impaired right disc mobility, symmetric disc closure, and a hypodense mass (arrowhead in panel **b**) adjacent to the sewing ring

Cardiac CT was performed in 65 patients with suspected PVD. No patients were excluded due to insufficient image quality. One patient was excluded due to unavailable clinical follow-up, resulting in a study population of 64 patients (age 58.6 ± 16.9 years; 27 women, 42.2%). PHVs were mechanical (44, 68.8%), bioprosthetic (12, 18.8%), and transcatheter (8, 12.5%), and located in aortic (47, 73.4%), mitral (15, 23.4%), and pulmonic (2, 3.1%) positions. The time interval between PHV implantation and CT evaluation was 7.7 ± 7.0 years. Baseline characteristics are presented in Tab. 1. In addition, representative cases are presented in Figs. 1 and 2.

**Table 1:** Baseline of the entire cohort with possible PVD

Characteristics	All (*n* = 64)
Age, years	58.6 (16.9)
Female sex, *n* %	27 (42.2)
*Left ventricular ejection fraction*	
– Normal (> 55%)	45 (70.3)
– Mildly reduced (41%–54%)	13 (20.3)
– Moderately reduced (30–40%)	2 (3.1)
– Severely reduced (< 30%)	4 (6.3)
Age at time of implantation (year)	50.7 (18.7)
*Valve type*	
– Mechanical	44 (68.8)
– Biological	12 (18.8)
– Transcatheter	8 (12.5)
*Valve Position*	
– Aortic	47 (73.4)
– Mitral	15 (23.4)
– Pulmonic	2 (3.1)
*Indication for CT*	
– Dyspnoea	22 (34.4)
– Infection	10 (15.6)
– Cerebrovascular stroke	9 (14.1)
– High gradient (during follow up)	15 (23.4)
– Heart Failure	8 (12.5)
*Echocardiographic findings*	
– Normal valve function	23 (35.9)
– Suspected stenosis	27 (42.2)
– Significant stenosis	14 (21.9)
*PVD etiology on CT*	
– Pannus	10 (15.6)
– Thrombus	6 (9.4)
– Vegetations	3 (4.7)
– Prosthetic valve malposition (transcatheter PHV)	2 (3.1)
– Structural valve deterioration (biological PHV)	2 (3.1)
– None	41 (64.1)
Limited leaflet motion (in mechanical PHV)	18 (28.1)

Categorical variables are represented as counts with percentages, and normally distributed continuous variables are represented as means with standard deviations

*CT* computed tomography, *PHV* prosthetic heart valve, *PVD* prosthetic valve dysfunction

### Negative CT findings

Of the 64 patients, 34 (53.1%) had negative CT findings without structural abnormalities. During a follow-up of 3.5 ± 1.8 years, 2 patients (5.8%) reached the primary endpoint, both due to endocarditis not detected on CT but confirmed intraoperatively within 5 months. One case involved an abscess surrounding a Carpentier-Edwards Magna Ease 23 mm bioprosthesis (Fig. S2), and the other involved a paravalvular leak adjacent to a mechanical Carbomedics 25 mm aortic valve. One additional case of endocarditis occurred > 3 years after CT and was therefore considered unrelated. The NPV of CT was 94% for ruling out the primary endpoint within 1 year.

### Positive CT findings

Among the 30 (46.9%) patients with abnormal findings, a periprosthetic mass was identified in 21 (70%). Etiologies included pannus (10, 33.3%), thrombus (6, 20.0%), vegetations (3, 10.0%), and structural valve deterioration (2, 6.7%). In 2 patients (6.7%) CT demonstrated transcatheter PHV malposition. No cases of prosthesis–patient mismatch were observed.

Among the 23 patients with a mechanical PHV, 18 (78.3%) had restricted leaflet motion. A total of 11 of these 18 patients had an evident structural mass as the definitive cause. In the remaining 7 patients, no visible mass and no significant increase in gradients on echocardiography were observed.

Management included observation in 12 patients (40.0%), pharmacological treatment in 7 patients (23.3%), surgery in 10 patients (33.3%), and transcatheter valve implantation in 1 patient. In 7 of 10 surgical patients, CT findings were confirmed intraoperatively (pannus in 3, thrombus in 2, leaflet calcification in 1, and endocarditis in 1 patient). In the remaining 3 patients, restrictive leaflet motion was present on CT but without identifiable morphological abnormalities seen intraoperatively. Two patients with a mechanical Carbomedics 25 mm aortic valve and recurrent cerebrovascular events showed restricted leaflet motion on CT without an identifiable structural mass; surgery revealed a washed-out abscess in one case and large thrombi in the other one. One patient with double mechanical valves (Carbomedics 19 mm mitral, 27 mm aortic) and dyspnea showed restricted aortic valve opening on CT without pannus or thrombus; surgery revealed a fibrotic ring that caused obstruction. The PPV of CT for detecting the underlying pathology was 70%.

### Transthoracic echocardiographic findings

**Fig. 3:**
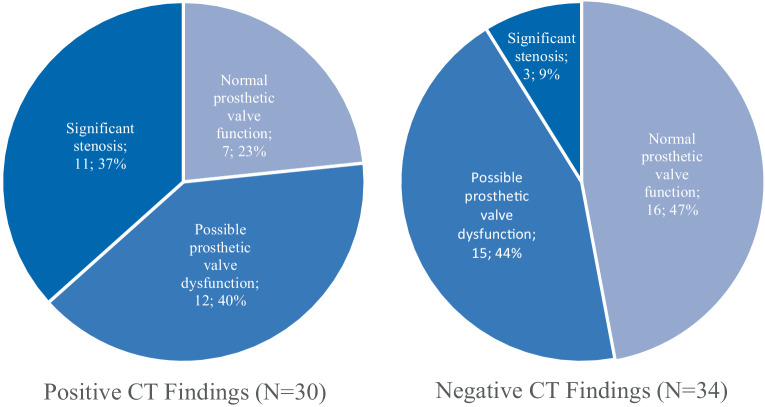
Transthoracic echocardiography findings in patients with positive and negative CT findings regarding PVD. Echocardiography performed within 3 months of CT categorized valve function as normal, as suggestive of a stenosis, or as a significant stenosis. The distribution of these findings is displayed for the CT-positive and CT-negative patient groups, showing a significant between-group difference (*p* = 0.004)

TTE was performed in all patients within 3 months of CT. *Normal prosthetic valve function* was observed in 23 patients (35.9%), *possible dysfunction *in 27 (42.2%), and *significant stenosis* in 14 (21.9%). The prevalence of possible valve dysfunction on TTE was comparable between CT-negative and CT-positive patients (44.1% vs. 40.0%), while overall distribution differed significantly between the two groups (*p* = 0.004) (Fig. 3). Among 20 bioprosthetic valves, 8 had no aortic insufficiency, 10 mild and 1 moderate. Among 10 surgical patients, TTE showed significant stenosis in 4, possible stenosis in 3, and normal function in 1. In 3 of these 10 cases, TTE correctly identified underlying pathology confirmed intraoperatively (thrombus-like structure in 1, and leaflet fibrosis or calcification in 2) (Fig. S3).

## Discussion

### Main findings

This cardiac CT study investigated 64 patients with suspected PVD, including 44 patients with mechanical PHV. Over a mean follow-up of 3.5 ± 1.8 years, only 2 of all 34 patients *without* CT abnormalities showed PVD-related adverse events, resulting in a high NPV of 94%. Among patients *with* CT abnormalities, a definitive PVD etiology (pannus, thrombus, and structural abnormalities) was identified in 78%. In 7 of all 10 patients who underwent surgical valve replacement, intraoperative findings confirmed the CT-detected abnormalities, resulting in a 70% PPV for detecting the underlying pathology of PVD.

### Previous studies

Previous studies on cardiac CT in PVD have mainly focused on its diagnostic value in identifying the pathophysiological mechanism, often in relatively small cohorts [7, 14–21]. A meta-analysis reported higher sensitivity for detecting pannus with CT than with TEE (85% vs 62%), whereas the sensitivity for thrombus was higher with TEE than with CT (90% vs 75%). While this study included 229 patients, only a small subset of approximately 50 patients underwent MDCT with surgical confirmation [7]. Thus, the diagnostic role of CT is increasingly recognized, whereas its prognostic value and its capacity to reliably exclude valve pathology remain less well defined [17].

A recent retrospective single-center study by Sandhu et al. evaluated 132 patients with PVD using TTE and cardiac CT [17]. During a mean follow-up of 1.7 years, 65% reached the composite endpoint of reoperation or all-cause mortality. The NPV of CT was 62%, while the PPV was 86%. Notably, their cohort predominantly consisted of *bioprosthetic* PHVs (97/132, 73%), with structural valve degeneration and leaflet calcification as the most frequent findings; in addition, 31 mechanical and 4 transcatheter valves were assessed. The lower NPV could reflect a limited ability of CT to assess functional prosthetic regurgitation, which was present in 35% of their patients. This highlights that CT for *bioprosthetic *PHVs should be interpreted as a complementary modality: echocardiography remains essential for assessing hemodynamic consequences whereas CT provides detailed anatomical insight into the underlying mechanism. In contrast, our cohort consisted mainly of *mechanical *PHVs (44/64, 69%), a subgroup in which CT allows both structural and functional assessment through precise measurement of the leaflet opening and closing angles. Only 2 patients (NPV 94%) with normal CT findings experienced adverse clinical events, supporting the value of CT for ruling out mechanical PVD.

Our findings are consistent with previous data comparing cardiac CT findings with intraoperative findings. Teshima et al. used CT to analyze 13 patients with aortic PHV obstruction, of whom only 2 proceeded to surgery; pannus was confirmed in both [25]. Symersky et al. studied 18 patients with acquired PHV obstruction of unknown cause, of whom 8 mechanical PHVs were reoperated on; thrombus and/or pannus was surgically confirmed in 5 cases, all of which had been identified on CT [26]. Ueda et al. reported on 9 patients with acquired mechanical aortic PHV obstruction in whom echocardiography did not reveal masses. However, CT demonstrated subvalvular tissue with an anatomical configuration consistent with pannus, subsequently confirmed at surgery in all cases [27]. More recently, Ikram et al. used cardiac CT to evaluate 54 patients with suspected PHV dysfunction [4]. In their cohort, dyspnea (52%) was the most common symptom in patients with suspected PHV dysfunction, while one third of all patients with findings suspicious for PVD was identified during routine echocardiographic follow-up in the absence of symptoms (32%). CT revealed *obstructive* PVD in 78% of the cases, while stenosis severity was not reported. CT was found to be superior to TTE in identifying pannus (in 12 versus 7 patients). In 5 of all 6 patients who underwent surgery, intraoperative findings confirmed the CT diagnoses: 2 cases with pannus formation; 2 with thrombotic obstruction; and 1 with infective endocarditis [4].

In this study, Agatston calcium scoring was used to quantify calcific degeneration. Although this provides an objective parameter, its interpretability remains limited by the lack of validated thresholds for bioprosthetic valves and its inability to detect fibrotic degeneration. Nevertheless, it may improve standardisation if validated in larger cohorts.

### Clinical implications

In daily clinical practice, suspicion for PVD is typically based on symptoms and echocardiographic findings. Our results suggest that CT provides important incremental value both in confirming and ruling out PVD. Most patients without CT abnormalities remained free from adverse clinical events during follow-up, supporting its potential role in reducing unnecessary diagnostic testing, such as repeated echocardiography and fluoroscopy. Despite its diagnostic advantages, CT has limitations, particularly in ruling out prosthetic valve infection that may still be in its earliest, subclinical stages. Small, mobile vegetations may be missed, and echocardiography remains essential [19, 22, 28]. In our study, paravalvular complications were identified at surgery months after the initial CT, which may have developed after imaging, yet it is also plausible that they were already present in a very early form and not recognised on CT.

Discrepancies between CT and TTE highlight the complementary nature of both modalities. CT detected abnormalities in 28% of patients with normal TTE, including subtle leaflet restriction without elevated gradients. However, the > 4° threshold used to define abnormal leaflet motion on CT is based on manufacturer reference values and may not always indicate haemodynamic consequences and clinically relevant dysfunction. Therefore, optimal evaluation of suspected valve dysfunction requires integration of both imaging modalities, echocardiography and CT, as emphasized in previous studies and current international guidelines [5, 6, 23, 24].

### Strengths and limitations

This study includes one of the larger cohorts of patients with suspected PVD, predominantly with mechanical PHVs. The study provides insights into the diagnostic utility of CT for suspected PVD but also for ruling out PVD, both of which have a major impact on a patient’s clinical management and prognosis. Limitations include the retrospective design. In addition, statistical power was limited by the sample size and relatively low event rate, which may partly reflect the broad inclusion criteria based on both echocardiographic findings and clinical suspicion. CT protocols were applied at the discretion of the clinical team and generally represented a radiation-saving strategy (dose modulation and a 20–90% reconstruction window) without evident loss of diagnostic performance. The use of a composite reference standard may have introduced verification bias, as absence of events during follow-up was considered a true negative. In addition, not all CT findings could be surgically confirmed due to clinical constraints.

## Conclusion

This study is the first to demonstrate the high negative predictive value of CT for ruling out significant PVD-related complications, reinforcing its role as a reliable modality for ruling out major adverse events. While the positive predictive value for detecting the underlying pathology of PVD was moderate, CT effectively identified structural abnormalities that required intervention, which supports its clinical usefulness for the comprehensive assessment of suspected PVD.

## Supplementary Information

**Supplementary Fig. 1. How to distinguish thrombus from pannus formation on cardiac CT.** CT appearances of thrombus versus pannus in prosthetic heart valves with corresponding schematic illustrations. Panels A–B show thrombus on a Perimount Magna Mitral Ease bioprosthesis (29 mm), appearing as an irregular, asymmetric hypodense mass attached to the leaflet and stent frame. Panels C–D depict pannus beneath a Carbomedics Top Hat mechanical aortic prosthesis (25 mm). The slightly oblique imaging plane visualizes pannus as a thin, smooth, concentric hypodense rim directly underneath the valve ring.

**Supplementary Fig. 2. Abscess of a bioprosthetic aortic valve initially missed on CT.** Images from a patient with a Carpentier Edwards Magna Ease bioprosthetic aortic valve (25 mm) who presented with fever and suspected endocarditis lenta due to Propionibacterium acnes due to new conduction abnormalities, there was a high suspicion of periannular involvement. Cardiac CT, including dedicated late venous phase imaging, demonstrated subtle early periannular changes yet no rim enhancement and therefore did not identify an abscess (arrows, Panels A and B). Four days later, transoesophageal echocardiography confirmed a periannular abscess and vegetation (arrows and circle, Panels C and D). Two months later, after antibiotic therapy, valve dehiscence and an abscess were confirmed during surgery.

**Supplementary Fig. 3. Severe structural degeneration of a Freestyle bioprosthesis with a markedly elevated Agatston score. **Images from an adolescent patient with a Freestyle bioprosthetic valve (29 mm) presenting with progressive exercise intolerance. Compared with one year earlier, transthoracic echocardiography showed increased transvalvular gradients, consistent with moderate low-flow, low-gradient aortic stenosis (max/mean gradient 18/10 mm Hg, AVA 1.3 cm^2^, dimensionless index 0.28). Transesophageal echocardiography (Panels A and B) showed poor leaflet motion. Cardiac CT (Panel C) revealed diffuse leaflet thickening with fibrotic and calcific changes. Although the effective orifice area was 1.9 cm^2^ (Panel D), the Agatston calcium score was elevated at 2550 AU, indicating advanced degeneration. Additional exercise testing showed a failure of blood pressure to rise appropriately. The patient underwent surgery, and findings confirmed the Freestyle prosthesis was severely calcified, consistent with the CT findings of advanced structural valve degeneration.

## Data Availability

The data underlying this article are available from the corresponding author upon reasonable request, subject to institutional approval and applicable privacy regulations.
