# Supplementary figures and images for: Clinical value of computed tomography in suspected prosthetic valve dysfunction
Source: Neth Heart J. 2026 Jul 2;34(7-8):265–73. doi: 10.1007/s12471-026-02052-8 (PMC13376089; doi:10.1007/s12471-026-02052-8)

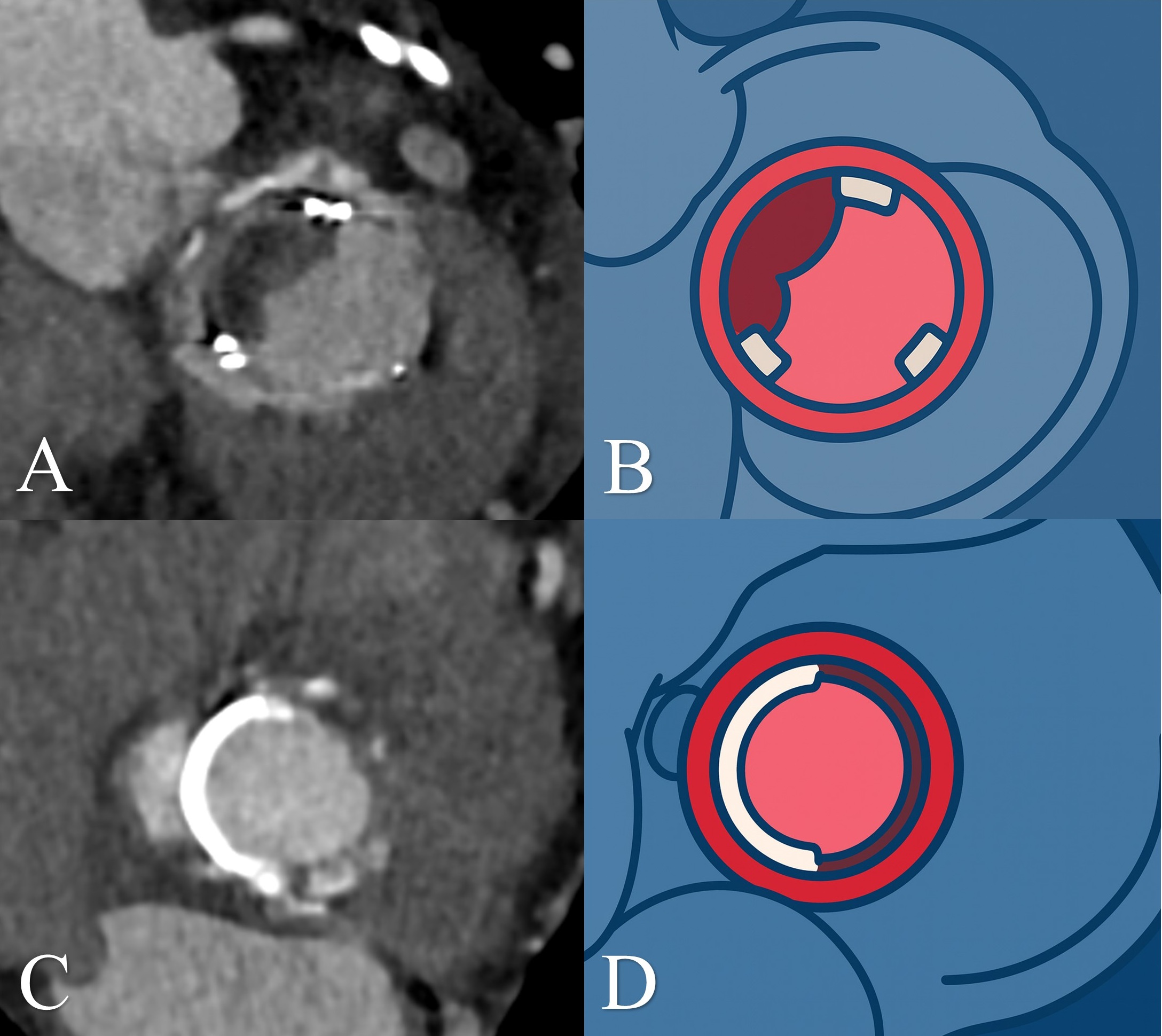

Supplement: Supplementary file 1 — Supplementary Fig. 1. How to distinguish thrombus from pannus formation on cardiac CT. CT appearances of thrombus versus pannus in prosthetic heart valves with corresponding schematic illustrations. Panels A–B show thrombus on a Perimount Magna Mitral Ease bioprosthesis (29 mm), appearing as an irregular, asymmetric hypodense mass attached to the leaflet and stent frame. Panels C–D depict pannus beneath a Carbomedics Top Hat mechanical aortic prosthesis (25 mm). The slightly oblique imaging plane visualizes pannus as a thin, smooth, concentric hypodense rim directly underneath the valve ring. [file 12471_2026_2052_MOESM1_ESM.jpg]

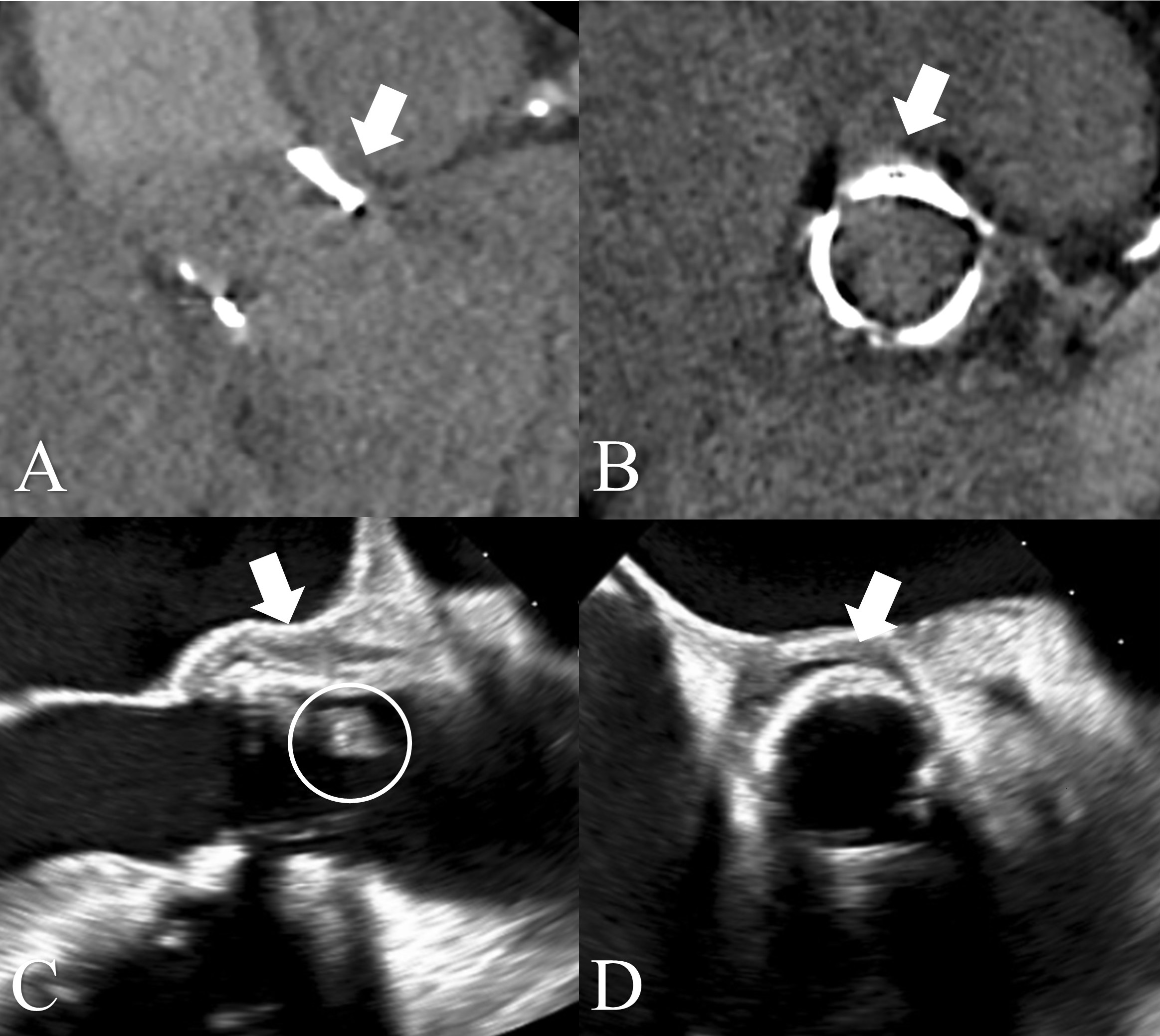

Supplement: Supplementary file 2 — Supplementary Fig. 2. Abscess of a bioprosthetic aortic valve initially missed on CT. Images from a patient with a Carpentier Edwards Magna Ease bioprosthetic aortic valve (25 mm) who presented with fever and suspected endocarditis lenta due to Propionibacterium acnes due to new conduction abnormalities, there was a high suspicion of periannular involvement. Cardiac CT, including dedicated late venous phase imaging, demonstrated subtle early periannular changes yet no rim enhancement and therefore did not identify an abscess (arrows, Panels A and B). Four days later, transoesophageal echocardiography confirmed a periannular abscess and vegetation (arrows and circle, Panels C and D). Two months later, after antibiotic therapy, valve dehiscence and an abscess were confirmed during surgery. [file 12471_2026_2052_MOESM2_ESM.jpg]

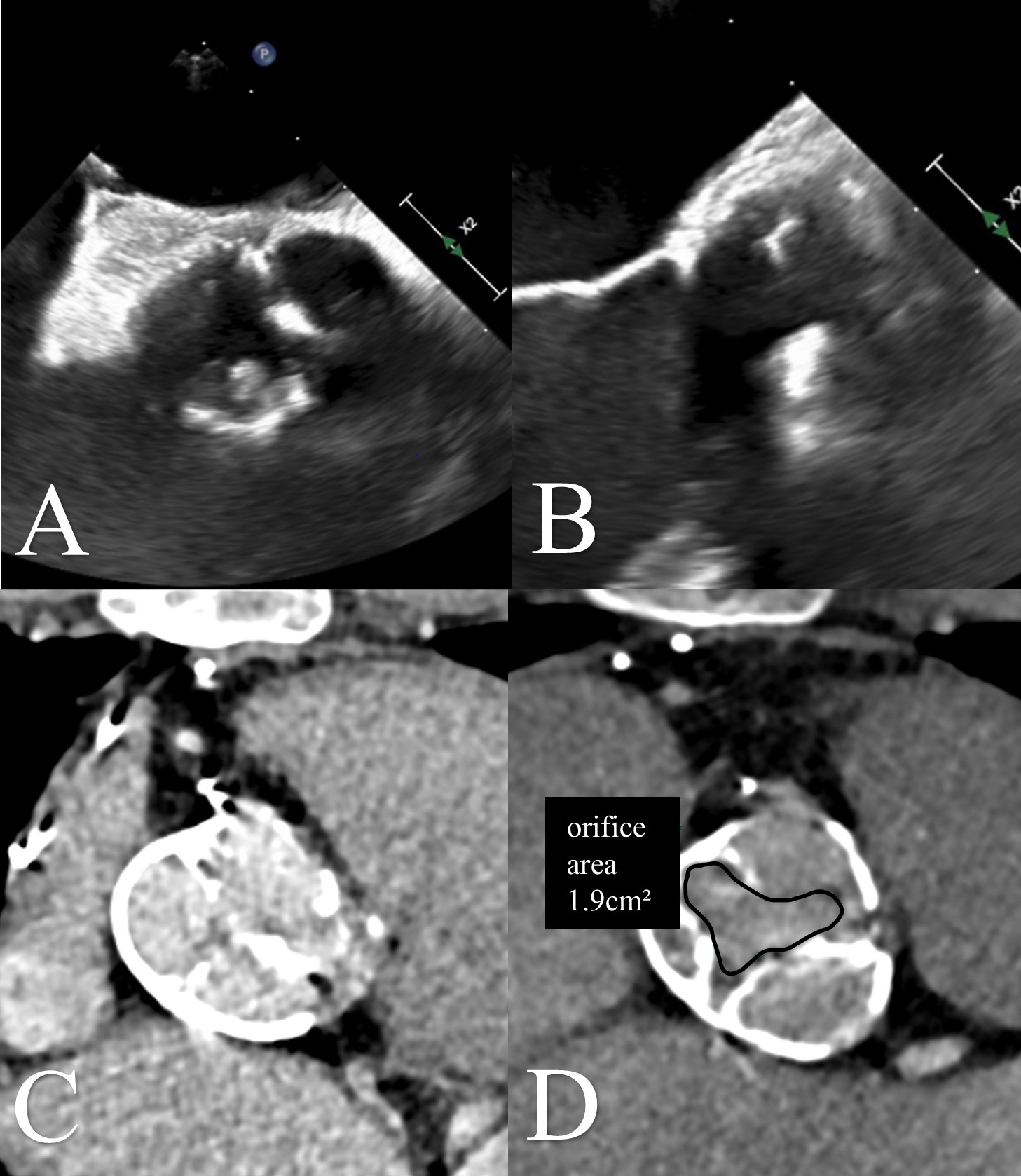

Supplement: Supplementary file 3 — Supplementary Fig. 3. Severe structural degeneration of a Freestyle bioprosthesis with a markedly elevated Agatston score. Images from an adolescent patient with a Freestyle bioprosthetic valve (29 mm) presenting with progressive exercise intolerance. Compared with one year earlier, transthoracic echocardiography showed increased transvalvular gradients, consistent with moderate low-flow, low-gradient aortic stenosis (max/mean gradient 18/10 mm Hg, AVA 1.3 cm2, dimensionless index 0.28). Transesophageal echocardiography (Panels A and B) showed poor leaflet motion. Cardiac CT (Panel C) revealed diffuse leaflet thickening with fibrotic and calcific changes. Although the effective orifice area was 1.9 cm2 (Panel D), the Agatston calcium score was elevated at 2550 AU, indicating advanced degeneration. Additional exercise testing showed a failure of blood pressure to rise appropriately. The patient underwent surgery, and findings confirmed the Freestyle prosthesis was severely calcified, consistent with the CT findings of advanced structural valve degeneration. [file 12471_2026_2052_MOESM3_ESM.jpg]
